# Supplementary material for: Prevalence of scabies and associated factors among children aged 5–14 years in Meta Robi District, Ethiopia
Source: PLoS One. 2023 Jan 3;18(1):e0277912. doi: 10.1371/journal.pone.0277912 (PMC9810185; doi:10.1371/journal.pone.0277912)
Supplement: S1 File — (DOCX) [file pone.0277912.s002.docx]

**Annex I- Questionnaires in English Version**

**Consent**

Dear, Hello! My name is ___________________. I’m data collector for the study to determine prevalence of scabies and associated factors. You are kindly requested to be included in the study, which will have importance in improving scabies prevalence and its associated factors. The interview will take about 15-20 minutes. The study has approval from Wollega University Institute of Health science Research, Technology Transfer and Graduate study associated Office. The information that is obtained from this study will be used for research purpose only and your privacy & confidentiality were secured throughout the study. So I politely request your cooperation to respond to my questionnaire. You do have the right not to respond at all or to withdraw in the meantime, but your participation has great value for the success of this study objective.

Do you agree to continue the interview? Yes No

Thank you for your cooperation!

**Background information**

District __________________________ Kebele __________________Zone ___________

Name of Interviewer ________________Interview date___/______/_____signature_______

Name of Supervisor ________________Supervised date____/______/____signature______

**Part I. Socio demographic information of parent/care givers respondents of Meta Robi**

**District, West Shoa Zone, Oromia, Ethiopia, 2021**

| **S/N** | **Socio demographic data** | **Possible choices/Answers** | **Remark** |
| --- | --- | --- | --- |
|  | Respondent’s position in the household | 1. Husband 2. Wife 3. Child 4. Relative 5. Other specify____________________ |  |
|  | Respondent’s Sex | 1. Male 2. Female |  |
|  | Age of respondent | _______________/year/ |  |
|  | Religion | 1. Protestant 2. Orthodox 3. Wakefeta 4. Muslim 5. Catholic 6. Other (specify)_________________ |  |
|  | Ethnicity | 1. Oromo 2.Amhara  3. Gurage 4. SNNP  5. Others (specify) __________ |  |
|  | Marital status | 1. Single 2. Married  3. Divorced 4. Widowed  5. Cohabitating |  |
|  | Educational status | 1. Unable to read and write  2. Only Read & Write  3. Primary school (1-8) 5.Secondary school (9-12) 6.College or university |  |
|  | Occupation | 1. Farmer  2. House wife  3.Merchant  4. Government employee  5. Daily labor  6. Student  7. Unemployed  8. Other (specify)___________________ |  |
|  | Number of Family Size | __________ (total male and female) |  |
|  | Average annually income  of the family | ___________( ETB ) |  |
|  | Are/is there children aged 5-14 year in the household? | 1. Yes  2. No |  |
|  | If yes to Q111 Number of children aged 5-14 year in the household | _______(in number) |  |
|  | Have you ever heard about the scabies? | 1. Yes  2. No |  |
|  | If yes to Q 113 from where do you heard about scabies? | 1. Radio 2. Television 3. Health worker 4. Other (specify)_________________ |  |
|  | Do you know sign and symptoms of scabies? | 1. Yes 2. No |  |
|  | If yes to Q 115 What are the sign and symptoms of scabies? | 1. Itching 2. Rash 3. Burrowing 4. Other specify _________________ |  |
|  | Is scabies transmitted from person to person? | 1. Yes  2. No |  |
|  | If yes to Q117 how can it be transmitted? | 1. Skin to skin contact 2. Sharing contaminated clothes and beds 3. Others(specify) __________________ |  |
|  | Is Scabies preventable? | 1. Yes  2. No |  |
|  | If yes to Q119 how can it be prevented? | 1. Treatment 2. avoid skin contact 3. avoid sharing of cloths and beds 4. avoid over crowding 5. improve hygiene and sanitation 6. Others(specify) __________________ |  |

**Part II. Environmental and WASH related factors of study respondents of Meta Robi District, West Shoa Zone, Oromia, Ethiopia, 2021**

| **S/N** | **WASH and Environmental data** | **Possible choices/Answers** | **Remark** |
| --- | --- | --- | --- |
|  | What water sources do you use? | 1. Pipe 2. Protected well 3. Protected spring 4. Unprotected spring 5. Unprotected well |  |
|  | Water consumption in liters/ day/ house hold? | _______ ( liter/ house hold/day ) |  |
|  | How much time it takes to fetch water? | 1. Less than 30 minutes 2. 30-60 minutes 3. More than 60 minutes |  |
|  | How often do you wash your cloth? | 1. once a month 2. twice a month 3. three times per a month 4. four times per a month 5. more than four per a month |  |
|  | How often do you take a bath? | 1. Once a week 2. Twice a week 3. Three times per week 4. Monthly 5. More than month |  |
|  | When do you wash your hand? | 1. Before meal 2. After meal 3. Before breast feeding 4. After using latrine 5. Before food preparation 6. After touching contaminated objects 7. Others(specify)______________ ____________________ | Multiple response is possible |
|  | What do you use to wash your hand? | 1. Water only 2. Water and soap 3. water and ash 4. Water and alcohol/sanitizer |  |
|  | Do your family members share clothes? | 1. Yes 2. No |  |
|  | Do your families share clothes with other relative/friends? | 1. Yes 2. No |  |
|  | Do all family members share bed/sleeping place? | 1. Yes 2. No |  |
|  | How many rooms does your house have? | 1. One 2. Two 3. Three 4. Four 5. More than four |  |

**Part III. Children and family related factors of the study participants in Meta Robi District, West Shoa Zone, Oromia Regional State, Ethiopia, 2021**

| SN | Children and family data | **Possible choices/Answers** | **Remark** |
| --- | --- | --- | --- |
|  | How often do children aged 5-14 year wash his/her cloths? | 1. Once a month 2. Twice a month 3. Three times per a month 4. Four times per a month 5. More than four per a month |  |
|  | How often do children aged 5-14 year take a bath? | 1. Once a week 2. Twice a week 3. Three times per week 4. Monthly 5. More than month |  |
|  | When do children age 5-14 year wash his/her hand? | 1. Before meal 2. After meal 3. After using the toilet 4. After touching contaminated objects 5. After hand shaking 6. Others(specify)__________________________ |  |
|  | What do children aged 5-14 year use to wash his/her hand? | 1. Water only 2. Water and soap 3. water and ash 4. water and alcohol /sanitizer |  |
|  | Does children age 5-14 year share clothes with his/her family members? | 1. Yes 2. No |  |
|  | Does children age 5-14 year share bed/ sleeping place with his/her family members? | 1. Yes 2. No |  |
|  | How many family member share sleeping place/clothes? | _______(Number) |  |
|  | Do any of family members have history of itch rash/scabies past two months? | 1. Yes 2. No |  |
|  | Do children aged 5-14 year have contact history with scabies cases past two months? | 1. Yes 2. No |  |
|  | Do children age 5-14 year share clothes with friends/relative other than family members? | 1. Yes 2. No |  |

**Part IV. Clinical diagnosis of scabies among children aged 5-14 year in Meta Robi district based on IACS, 2020.**

| SN | Scabies status and demographic data | Responses | Remark |
| --- | --- | --- | --- |
|  | Current Sign and Symptom of study Children aged 5-14 year? | 1. Typical lesions in a typical distribution and two history features 2. Typical lesions in a typical distribution and one history feature 3. Atypical lesions or atypical distribution and two history feature 4. None 5. Other(specify) ______________ |  |
|  | Number of lesions | _______________________ |  |
|  | Locations of scabies | 1. Upper extermities 2. Lower extermities 3. Others (specify) 4. Others |  |
|  | Scabies status/Result of examination | 1. Positive (has clinical scabies)  2. Negative(has no clinical scabies) |  |
|  | Sex of children aged 5-14 year | 1. Male  2. Female |  |
|  | Age of children aged 5-14 year | _______ (in year) |  |
|  | Is the child attending the school? | 1. Yes 2. No |  |
|  | If yes to Q146, what is the number of students in their class? | _______(in number) |  |

**Part V. Health Service utilization related factors in Meta Robi District, West Shoa Zone, Oromia Regional State, Ethiopia, 2021**

| SN | Health Service utilization data | Responses | Remark |
| --- | --- | --- | --- |
|  | Is there a family member with history of scabies past two month? | 1. Yes 2. No |  |
|  | If “Yes” to Q148 do scabies cases was treated? | 1. Yes 2. No |  |
|  | If “Yes” to Q148 do all family members being treated? | 1. Yes 2. No |  |
|  | If “No” to Q148 why? | 1. Lack of money 2. Shortage of drugs from HF 3. No Health Facility around 4. Others ________________ |  |
|  | How much time it takes to reach to health facility? | ___________ (in hours) |  |
