# Supplementary material for: Prevalence of scabies and associated factors among children aged 5–14 years in Meta Robi District, Ethiopia
Source: PLoS One. 2023 Jan 3;18(1):e0277912. doi: 10.1371/journal.pone.0277912 (PMC9810185; doi:10.1371/journal.pone.0277912)
Supplement: Attachment — Submitted filename: Response to Reviewers.docx [file pone.0277912.s005.docx]

**Date: 25 October 2022**

To PLOS ONE Journal

From Werku Etafa, [witafay@gmail.com](mailto:witafay@gmail.com)

**Subject: Sending revised manuscript**

Dear Editor,

We greatly value the time and effort you scientifically monitored and evaluated our manuscript status entitled "Prevalence of scabies and associated factors among children aged 5-14 years in Meta Robi District, Ethiopia" (manuscript number: **PONE-D-22-15754R1**). All the comments were valuable and very constructive to improve our work. We checked all reference list are complete and correct. We found reference number 18 and 35 as the same. We removed the reference in the list number 35. We also checked for the presence of retracted articles and there is no retracted article used to the level of our skill. We have tried to address every comment carefully and made correction which I hope could the improved the quality of the manuscript. We also highly appreciated for reviewer's work, and revised our paper point-by-point as suggested.

Best Regards,

Werku Etafa, [witafay@gmail.com](mailto:witafay@gmail.com)

**Author's point-by-point response letter**

**Responses to Editor**

Dear editor,

We thank you for your constructive comments. Comments are marked in **BLACK** and responses are marked in RED color, pages referenced are from the final revised manuscript).

Comment: Please review your reference list to ensure that it is complete and correct. If you have cited papers that have been retracted, please include the rationale for doing so in the manuscript text, or remove these references and replace them with relevant current references.

Response: We carefully reviewed the entire reference list and it is complete and there is modification in sequencing. Reference number 18 and Reference number 35 were the same in the previously submitted manuscript. But, we avoided this duplication in the revised version of our manuscript. We checked all reference list and we could not find a retracted article.

Comment: Any changes to the reference list should be mentioned in the rebuttal letter that
accompanies your revised manuscript. If you need to cite a retracted article, indicate the article’s retracted status in the References list and also include a citation and full reference for the retraction notice.

Response: We included changes made in the reference list in the rebuttal letter. We did not use a retracted articles in the manuscript based on the level of our checking skill.
Responses to Reviewer
Comment: Dear author, no response was given to comments provided by the reviewer in line #30 and responses provided in line #201 is not clear and confusing. It is required to re-respond to these inquires before considering your work for publication.

Response: We apologize for our mistakes not providing responses to questions raised in the previous revision. For the question raised concerning line #30, we used a systematic type of random sampling (page 2, line 32).

Comment: Responses provided in line #201 is not clear and confusing.

Response: First, we communicated with the caregivers about the study and based on their willingness a written informed consent is obtained from the caregivers before they participate in the study (page 8, line 204-205)
